# 5-Chloro-2-methyl­sulfonyl-1,2,4-triazolo[1,5-*a*]quinazoline

**DOI:** 10.1107/S1600536812021770

**Published:** 2012-05-19

**Authors:** Rashad Al-Salahi, Mohamed Al-Omar, Mohamed Marzouk, Seik Weng Ng

**Affiliations:** a Department of Pharmaceutical Chemistry, College of Pharmacy, King Saud University, Riyadh 11451, Saudi Arabia; b Department of Chemistry, University of Malaya, 50603 Kuala Lumpur, Malaysia; c Chemistry Department, Faculty of Science, King Abdulaziz University, PO Box 80203 Jeddah, Saudi Arabia

## Abstract

The triazoloquinazole fused-ring system of the title compound, C_10_H_7_ClN_4_O_2_S, is essentially planar (r.m.s. deviation = 0.009 Å). In the methyl­sulfonyl substituent, the two S—O bonds are of equal length [1.402 (2) Å]. In the crystal, adjacent mol­ecules inter­act weakly through Cl⋯N contacts [*ca* 3.197 (2) Å].

## Related literature

For the synthesis of the precursor, see: Al-Salahi & Geffken (2011 ▶).
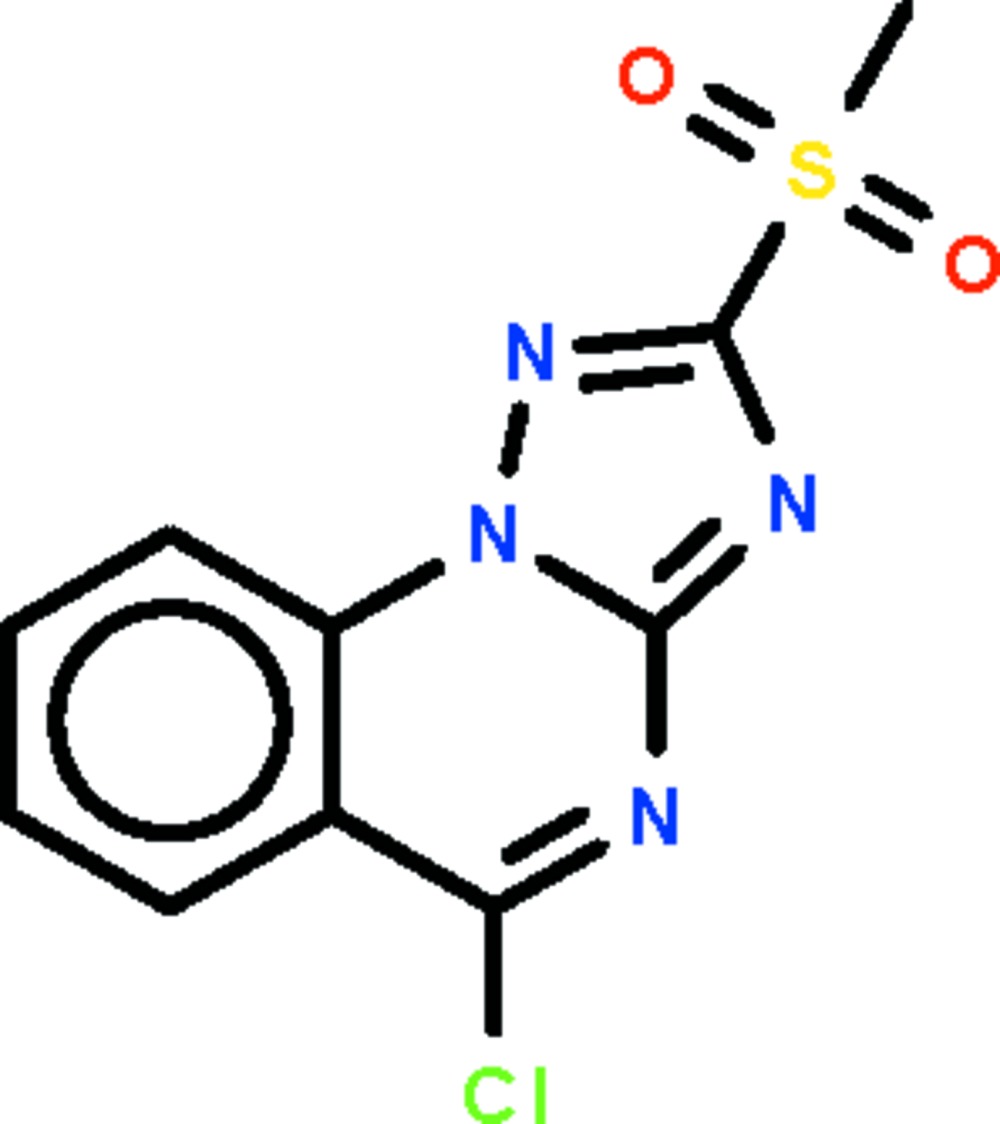


## Experimental

### 

#### Crystal data

C_10_H_7_ClN_4_O_2_S
*M* *_r_* = 282.71
Monoclinic, 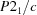

*a* = 12.6386 (3) Å
*b* = 10.7464 (3) Å
*c* = 8.6317 (3) Å
β = 102.459 (3)°
*V* = 1144.74 (6) Å^3^
*Z* = 4
Cu *K*α radiation
μ = 4.69 mm^−1^
*T* = 294 K
0.30 × 0.25 × 0.20 mm

#### Data collection

Agilent SuperNova Dual diffractometer with an Atlas detector
Absorption correction: multi-scan (*CrysAlis PRO*; Agilent, 2012) ▶ *T*_min_ = 0.334, *T*_max_ = 0.454
10130 measured reflections
2383 independent reflections
2168 reflections with *I* > 2σ(*I*)
*R*_int_ = 0.025

#### Refinement

*R*[*F* ^2^ > 2σ(*F* ^2^)] = 0.037
*wR*(*F* ^2^) = 0.113
*S* = 1.03
2383 reflections
165 parameters
H-atom parameters constrained
Δρ_max_ = 0.27 e Å^−3^
Δρ_min_ = −0.40 e Å^−3^

### 

Data collection: *CrysAlis PRO* (Agilent, 2012 ▶); cell refinement: *CrysAlis PRO*; data reduction: *CrysAlis PRO* program(s) used to solve structure: *SHELXS97* (Sheldrick, 2008 ▶); program(s) used to refine structure: *SHELXL97* (Sheldrick, 2008 ▶); molecular graphics: *X-SEED* (Barbour, 2001 ▶); software used to prepare material for publication: *publCIF* (Westrip, 2010 ▶).

## Supplementary Material

Crystal structure: contains datablock(s) global, I. DOI: 10.1107/S1600536812021770/bt5916sup1.cif

Structure factors: contains datablock(s) I. DOI: 10.1107/S1600536812021770/bt5916Isup2.hkl

Supplementary material file. DOI: 10.1107/S1600536812021770/bt5916Isup3.cml

Additional supplementary materials:  crystallographic information; 3D view; checkCIF report

## Figures and Tables

**Fig. 1.**
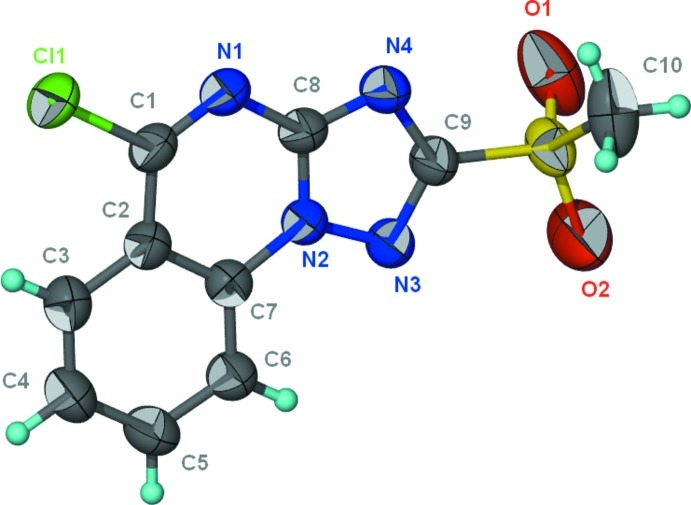
Anisotropic displacement ellipsoid plot (Barbour, 2001) of C10H7ClN4O2S at the 70% probability level; hydrogen atoms are drawn as spheres of arbitrary radius.

## supplementary crystallographic information

### Comment

In this study, 2-(methylsulfanyl)-[1,2,4]triazolo[1,5-*a*]quinazolin-5-one (Al-Salahi & Geffken, 2011) was first treated with phosphorus oxychloride to yield the chlorinated compound, whose sulfur linkage was then oxidized by hydrogen peroxide. Chlorination took place at the carbon atom bearing the ketonic oxygen in the title compound (Scheme I). The triazoloquinazole fused-ring system is planar; in the methanesulfonyl substitutent, the two S–O bonds are of equal length (1.402 (2) Å). Adjacent molecules interact weakly through Cl···N contacts (*ca*. 3.20 Å).

### Experimental

Under ice-cold conditions, 2-hydrazinobenzoic acid (10 mmol, 1.52 g) was added to a solution of dimethyl *N*-cyanodithioimidocarbonate (10 mmol, 1.46 g) in ethanol (20 ml). Triethylamine (30 mmol, 3.03 g) was added. The reaction mixture was stirred overnight at room temperature. Concentrated hydrochloric acid was added; the acidified mixture for heated for an hour. The mixture was poured into ice water; the solid that formed was collected and recrystallized from ethanol to give colorless crystals of 2-(methylsulfanyl)-[1,2,4]triazolo[1,5-*a*]quinazolin-5-one. The procedure was that reported earlier (Al-Salahi & Geffken, 2011).

The above compound (1 mmol, 0.232 g) was heated with phosphorus oxychloride (1 ml) in benzene (10 ml) for 2 h. The solvent was evaporated and the residue was treated with saturated potassium carbonate to give the chlorinated [1,2,4]triazoloquinazoline.

To the boiling mixture of chlorinated [1,2,4]triazoloquinazoline (1 mmol, 0.25 g) in glacial acetic acid (5 ml) was added hydrogen peroxide. Colorless crystals of the oxidized product were obtained when the solution was allowed to cool.

### Refinement

Carbon-bound H-atoms were placed in calculated positions [C–H 0.93 to 0.96 Å, *U*_iso_(H) 1.2 to 1.5*U*_eq_(C)] and were included in the refinement in the riding model approximation.

### Crystal data

**Table:**

C_10_H_7_ClN_4_O_2_S	*F*(000) = 576
*M**_r_* = 282.71	*D*_x_ = 1.640 Mg m^−^^3^
Monoclinic, *P*2_1_/*c*	Cu *K*α radiation, λ = 1.54184 Å
Hall symbol: -P 2ybc	Cell parameters from 4932 reflections
*a* = 12.6386 (3) Å	θ = 3.6–76.5°
*b* = 10.7464 (3) Å	µ = 4.69 mm^−^^1^
*c* = 8.6317 (3) Å	*T* = 294 K
β = 102.459 (3)°	Prism, colorless
*V* = 1144.74 (6) Å^3^	0.30 × 0.25 × 0.20 mm
*Z* = 4	

### Data collection

**Table:**

Agilent SuperNova Dual diffractometer with an Atlas detector	2383 independent reflections
Radiation source: SuperNova (Cu) X-ray Source	2168 reflections with *I* > 2σ(*I*)
Mirror monochromator	*R*_int_ = 0.025
Detector resolution: 10.4041 pixels mm^-1^	θ_max_ = 76.7°, θ_min_ = 3.6°
ω scan	*h* = −15→12
Absorption correction: multi-scan (*CrysAlis PRO*; Agilent, 2012)	*k* = −13→12
*T*_min_ = 0.334, *T*_max_ = 0.454	*l* = −10→10
10130 measured reflections	

### Refinement

**Table:**

Refinement on *F*^2^	Secondary atom site location: difference Fourier map
Least-squares matrix: full	Hydrogen site location: inferred from neighbouring sites
*R*[*F*^2^ > 2σ(*F*^2^)] = 0.037	H-atom parameters constrained
*wR*(*F*^2^) = 0.113	*w* = 1/[σ^2^(*F*_o_^2^) + (0.0707*P*)^2^ + 0.3878*P*] where *P* = (*F*_o_^2^ + 2*F*_c_^2^)/3
*S* = 1.03	(Δ/σ)_max_ = 0.001
2383 reflections	Δρ_max_ = 0.27 e Å^−^^3^
165 parameters	Δρ_min_ = −0.40 e Å^−^^3^
0 restraints	Extinction correction: *SHELXL97* (Sheldrick, 2008), Fc^*^=kFc[1+0.001xFc^2^λ^3^/sin(2θ)]^-1/4^
Primary atom site location: structure-invariant direct methods	Extinction coefficient: 0.0033 (6)

### Fractional atomic coordinates and isotropic or equivalent isotropic displacement parameters (Å^2^)

**Table:**

	*x*	*y*	*z*	*U*_iso_*/*U*_eq_	
Cl1	0.45911 (4)	0.43675 (5)	0.75576 (7)	0.05379 (19)	
S1	0.88914 (4)	0.86294 (4)	0.54205 (6)	0.04933 (19)	
O1	0.82482 (19)	0.94586 (18)	0.4366 (2)	0.0828 (7)	
O2	0.9760 (2)	0.8096 (2)	0.4887 (4)	0.1080 (10)	
N1	0.58990 (12)	0.60611 (16)	0.6966 (2)	0.0445 (4)	
N2	0.74566 (12)	0.56267 (13)	0.59166 (18)	0.0365 (3)	
N3	0.82959 (12)	0.62371 (15)	0.55072 (19)	0.0404 (3)	
N4	0.71600 (13)	0.75914 (15)	0.6396 (2)	0.0449 (4)	
C1	0.57202 (14)	0.48755 (18)	0.6911 (2)	0.0412 (4)	
C2	0.63661 (14)	0.39385 (17)	0.6353 (2)	0.0388 (4)	
C3	0.61415 (16)	0.26559 (18)	0.6285 (3)	0.0467 (4)	
H3A	0.5539	0.2353	0.6620	0.056*	
C4	0.68132 (17)	0.18528 (19)	0.5721 (3)	0.0518 (5)	
H4	0.6663	0.1005	0.5680	0.062*	
C5	0.77164 (17)	0.22883 (19)	0.5209 (3)	0.0498 (5)	
H5	0.8158	0.1727	0.4826	0.060*	
C6	0.79670 (16)	0.35396 (17)	0.5261 (2)	0.0431 (4)	
H6	0.8573	0.3829	0.4924	0.052*	
C7	0.72873 (14)	0.43547 (16)	0.5832 (2)	0.0368 (4)	
C8	0.67944 (14)	0.64414 (16)	0.6455 (2)	0.0394 (4)	
C9	0.80569 (15)	0.74008 (17)	0.5817 (2)	0.0408 (4)	
C10	0.9299 (2)	0.9347 (3)	0.7255 (3)	0.0708 (8)	
H10A	0.9786	1.0017	0.7171	0.106*	
H10B	0.9662	0.8752	0.8018	0.106*	
H10C	0.8676	0.9668	0.7591	0.106*	

### Atomic displacement parameters (Å^2^)

**Table:**

	*U*^11^	*U*^22^	*U*^33^	*U*^12^	*U*^13^	*U*^23^
Cl1	0.0401 (3)	0.0539 (3)	0.0736 (4)	−0.00281 (18)	0.0258 (2)	0.0039 (2)
S1	0.0629 (3)	0.0383 (3)	0.0549 (3)	−0.0106 (2)	0.0306 (2)	−0.00469 (19)
O1	0.1214 (18)	0.0528 (10)	0.0643 (11)	−0.0205 (10)	−0.0021 (11)	0.0108 (8)
O2	0.1003 (16)	0.0681 (13)	0.189 (3)	−0.0195 (11)	0.1040 (18)	−0.0260 (15)
N1	0.0364 (7)	0.0418 (8)	0.0590 (10)	0.0016 (6)	0.0187 (7)	−0.0013 (7)
N2	0.0350 (7)	0.0340 (7)	0.0427 (8)	−0.0004 (5)	0.0135 (6)	−0.0003 (6)
N3	0.0413 (8)	0.0373 (8)	0.0470 (8)	−0.0019 (6)	0.0194 (6)	−0.0006 (6)
N4	0.0438 (8)	0.0358 (8)	0.0602 (10)	0.0010 (6)	0.0224 (7)	−0.0011 (7)
C1	0.0319 (8)	0.0437 (10)	0.0497 (10)	0.0000 (7)	0.0126 (7)	0.0031 (8)
C2	0.0352 (8)	0.0378 (9)	0.0435 (9)	−0.0012 (7)	0.0088 (7)	0.0013 (7)
C3	0.0438 (9)	0.0404 (10)	0.0566 (11)	−0.0060 (8)	0.0124 (8)	0.0012 (8)
C4	0.0567 (12)	0.0351 (10)	0.0645 (13)	−0.0044 (8)	0.0154 (10)	−0.0030 (8)
C5	0.0534 (11)	0.0378 (10)	0.0606 (12)	0.0038 (8)	0.0177 (9)	−0.0055 (8)
C6	0.0418 (9)	0.0392 (9)	0.0512 (10)	0.0004 (7)	0.0165 (8)	−0.0006 (7)
C7	0.0361 (8)	0.0349 (9)	0.0396 (9)	0.0000 (6)	0.0082 (7)	0.0005 (7)
C8	0.0358 (8)	0.0372 (9)	0.0475 (10)	0.0026 (7)	0.0140 (7)	−0.0007 (7)
C9	0.0422 (9)	0.0357 (9)	0.0480 (10)	−0.0018 (7)	0.0176 (8)	−0.0005 (7)
C10	0.0859 (18)	0.0761 (17)	0.0471 (12)	−0.0406 (14)	0.0070 (11)	0.0010 (11)

### Geometric parameters (Å, º)

**Table:**

Cl1—C1	1.7288 (19)	C2—C3	1.406 (3)
S1—O1	1.402 (2)	C2—C7	1.408 (2)
S1—O2	1.402 (2)	C3—C4	1.371 (3)
S1—C10	1.737 (2)	C3—H3A	0.9300
S1—C9	1.7689 (19)	C4—C5	1.391 (3)
N1—C1	1.293 (3)	C4—H4	0.9300
N1—C8	1.363 (2)	C5—C6	1.380 (3)
N2—N3	1.357 (2)	C5—H5	0.9300
N2—C8	1.360 (2)	C6—C7	1.389 (3)
N2—C7	1.383 (2)	C6—H6	0.9300
N3—C9	1.327 (2)	C10—H10A	0.9600
N4—C8	1.324 (2)	C10—H10B	0.9600
N4—C9	1.349 (2)	C10—H10C	0.9600
C1—C2	1.443 (3)		
			
O1—S1—O2	115.62 (17)	C5—C4—H4	119.5
O1—S1—C10	109.07 (14)	C6—C5—C4	121.05 (19)
O2—S1—C10	112.43 (17)	C6—C5—H5	119.5
O1—S1—C9	108.29 (11)	C4—C5—H5	119.5
O2—S1—C9	107.48 (11)	C5—C6—C7	118.07 (18)
C10—S1—C9	103.11 (10)	C5—C6—H6	121.0
C1—N1—C8	115.74 (16)	C7—C6—H6	121.0
N3—N2—C8	110.54 (14)	N2—C7—C6	122.78 (17)
N3—N2—C7	125.77 (15)	N2—C7—C2	115.21 (16)
C8—N2—C7	123.68 (15)	C6—C7—C2	122.01 (17)
C9—N3—N2	100.26 (14)	N4—C8—N2	109.99 (16)
C8—N4—C9	101.57 (15)	N4—C8—N1	127.82 (17)
N1—C1—C2	126.35 (17)	N2—C8—N1	122.18 (16)
N1—C1—Cl1	116.69 (14)	N3—C9—N4	117.62 (16)
C2—C1—Cl1	116.95 (14)	N3—C9—S1	119.54 (14)
C3—C2—C7	118.11 (17)	N4—C9—S1	122.82 (14)
C3—C2—C1	125.06 (17)	S1—C10—H10A	109.5
C7—C2—C1	116.83 (16)	S1—C10—H10B	109.5
C4—C3—C2	119.81 (18)	H10A—C10—H10B	109.5
C4—C3—H3A	120.1	S1—C10—H10C	109.5
C2—C3—H3A	120.1	H10A—C10—H10C	109.5
C3—C4—C5	120.94 (19)	H10B—C10—H10C	109.5
C3—C4—H4	119.5		
			
C8—N2—N3—C9	−0.9 (2)	C3—C2—C7—C6	0.0 (3)
C7—N2—N3—C9	179.96 (17)	C1—C2—C7—C6	−179.65 (17)
C8—N1—C1—C2	−0.6 (3)	C9—N4—C8—N2	−0.5 (2)
C8—N1—C1—Cl1	179.85 (14)	C9—N4—C8—N1	179.41 (19)
N1—C1—C2—C3	−179.0 (2)	N3—N2—C8—N4	0.9 (2)
Cl1—C1—C2—C3	0.5 (3)	C7—N2—C8—N4	−179.93 (17)
N1—C1—C2—C7	0.7 (3)	N3—N2—C8—N1	−178.95 (16)
Cl1—C1—C2—C7	−179.81 (13)	C7—N2—C8—N1	0.2 (3)
C7—C2—C3—C4	0.0 (3)	C1—N1—C8—N4	−179.7 (2)
C1—C2—C3—C4	179.63 (19)	C1—N1—C8—N2	0.2 (3)
C2—C3—C4—C5	−0.2 (3)	N2—N3—C9—N4	0.7 (2)
C3—C4—C5—C6	0.4 (3)	N2—N3—C9—S1	−177.85 (13)
C4—C5—C6—C7	−0.3 (3)	C8—N4—C9—N3	−0.2 (2)
N3—N2—C7—C6	−1.7 (3)	C8—N4—C9—S1	178.32 (15)
C8—N2—C7—C6	179.27 (18)	O1—S1—C9—N3	122.07 (19)
N3—N2—C7—C2	178.86 (16)	O2—S1—C9—N3	−3.5 (2)
C8—N2—C7—C2	−0.1 (3)	C10—S1—C9—N3	−122.44 (19)
C5—C6—C7—N2	−179.21 (19)	O1—S1—C9—N4	−56.4 (2)
C5—C6—C7—C2	0.2 (3)	O2—S1—C9—N4	178.1 (2)
C3—C2—C7—N2	179.43 (17)	C10—S1—C9—N4	59.1 (2)
C1—C2—C7—N2	−0.2 (2)		
